# Supplementary material for: In Vitro Transfection of Up-Regulated Genes Identified in Favorable-Outcome Neuroblastoma into Cell Lines
Source: Cells. 2022 Oct 10;11(19):3171. doi: 10.3390/cells11193171 (PMC9564278; doi:10.3390/cells11193171)
Supplement: Supplementary file 1 [file cells-11-03171-s001.zip › cells-1888267-supplementary.pdf]

Supple. Table S1. Origins and characteristics of neuroblastoma cell lines.

Cell Line	Age/sex	INRG Stage	Origin	MYCN Status	p53 status	DHRS3 status	CYP26A1 status	NR0B1 status	Rethinol Sensitive	Cosmic ID
TGW	1y3m/Male	Unk	adrenal grand	Amplified	Mutation	Functional	Functional	Functional	+	COSS910780
SK-N-SH	4y/Female	M	thorax	Single	Functional	Functional	Functional	Functional	+	COSS717431
NH-12	1y3m/Male	M	adrenal grand	Amplified	Functional	Functional	Functional	Functional	+	COSS908447
NH-6	9m/Female	M	adrenal grand	Amplified	Functional	Functional	Functional	Functional	+	No
GOTO	1y1m/Male	M	adrenal grand	Amplified	Functional	Functional	Functional	Functional	Unknown	COSS906875
IMR-32	1y1m/Male	Unk	Abdomen	Amplified / DM	Functional	Unknown	Unknown	Unknown	+	No
SK-N-BE(2)	2y2m/Male	M	Unknown	Amplified	Mutation	Unknown	Unknown	Unknown	+	No

INRG: International neuroblastoma risk group, DM: double minutes

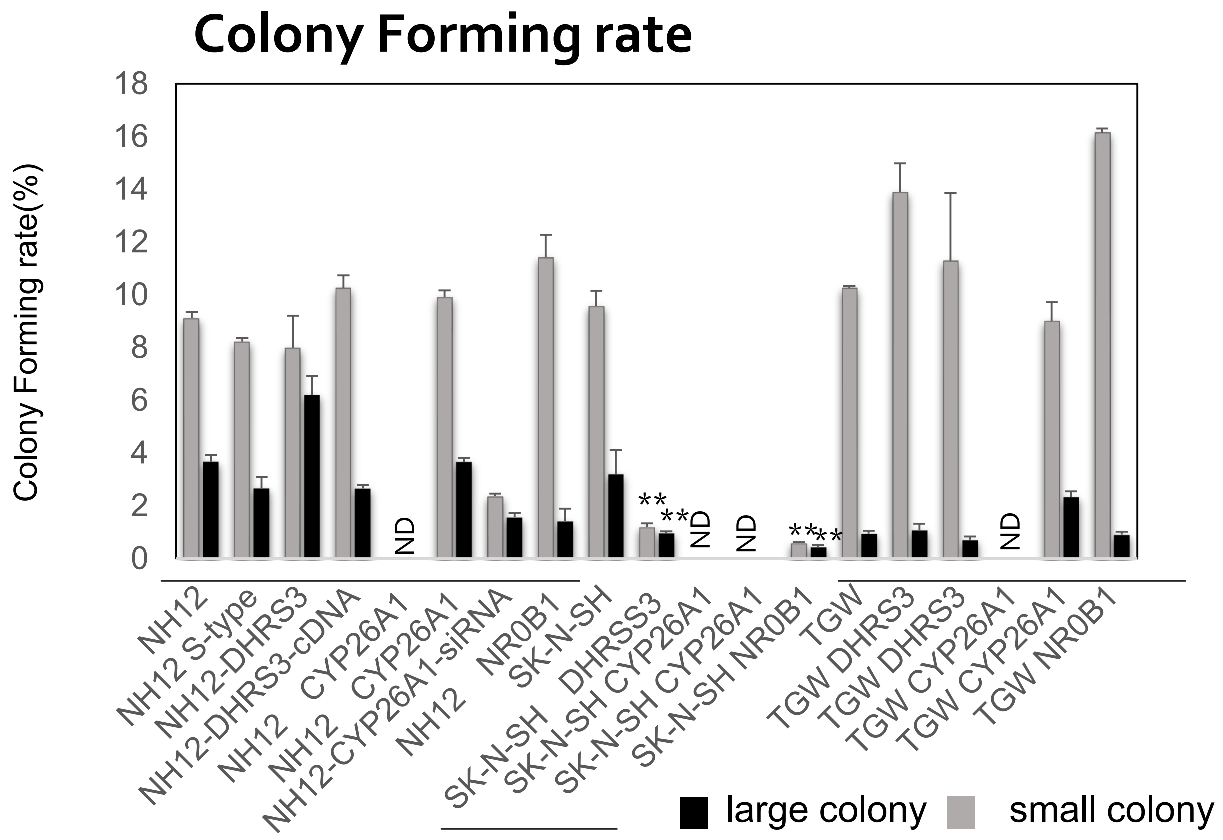

Supple. Figure S1. The colony rates in DHRS3-, CYP26A1-, and NROB1-overexpressing clones in soft-agar colony-formation assay.

Supple Table S2. Up- or Down- regulated pathway in DHRS3 expressing SK-N-SH cells.

a) Details of top 10 up-regulated pathways in DHRS3 expressing SK-N-SH

	Pathways (10 of 10)	p-value	Matched Entities	Pathway Entities	Gene
1	Hs_Vitamin_A_and_Carotenoid_Metabolism_WP716_83589	2.75E-04	2	43	Npc1l1, DHRS3
2	Hs_GPCR_ligand_binding_WP1825_83346	0.00175654	3	438	TAC3/TAC3, PRLHR/PRLHR
3	Hs_LGI-ADAM_interactions_WP3392_83198	0.006803198	1	12	LGI4
4	Hs_WNT_ligand_biogenesis_and_trafficking_WP2790_83226	0.014684311	1	26	WNT4 /N4GlycoAsn-WNT4 /N4GlycoAsn-PalmS WNT4
5	Hs_Signaling_by_Retinoic_Acid_WP3323_83286	0.023618052	1	42	DHRS3
6	Hs_Apoptotic_execution_phase_WP1784_83236	0.028055815	1	52	HIST1H1E
7	Hs_Arachidonic_acid_metabolism_WP2650_83044	0.029714996	1	53	FAAH2
8	Hs_DNA_Damage-Telomere_Stress_Induced_Senescence_WP3565_83455	0.03412625	1	61	HIST1H1E
9	Hs_Folate_Metabolism_WP176_82704	0.03687353	1	67	FOLR2
10	Hs_Visual_phototransduction_WP2776_83212	0.045614697	1	85	DHRS3

b) Details of top 10 down-regulated pathways in DHRS3 expressing SK-N-SH

	Pathways (10 of 46)	p-value	Matched Entities	Pathway Entities	Gene
1	Hs_Oncostatin_M_Signaling_Pathway_WP2374_73668	2.36E-05	5	65	PRKCB, OSMR, CYR61, CCL2, SERPINE1
2	Hs_Gastrin-CREB_signalling_pathway_via_PKC_and_MAPK_WP2664_83266	3.88E-06	8	180	PDKRB1,PDKRB2,HRH1,CYSLTR2, EDN1, EDN2,F2RL2,HBEGF
3	Hs_GPCR_ligand_binding_WP1825_83346	1.68E-05	11	438	BDKRB1,BDKRB2,CCL2, EDN1, EDN2, F2RL2(22-374), HRH1/HRH1, PTGER2, PTHLH, GPR39/GPR39
4	Hs_miRNA_targets_in_ECM_and_membrane_receptors_WP2911_83020	2.62E-05	4	45	
5	Hs_ACE_Inhibitor_Pathway_WP554_84372	9.02E-05	3	17	
6	Hs_Circadian_rythm_related_genes_WP3594_84573	9.58E-05	7	210	
7	Hs_TGF-beta_Receptor_Signaling_WP560_83868	1.99E-04	4	55	THBS1, SPP1, SERPINE1, LIF
8	Hs_Integrin_cell_surface_interactions_WP1833_83181	3.58E-04	4	66	THBS1, SPP1/SPP1, CD44/CD44, VCAM1/VCAM1
9	Hs_GPCRs,_Class_A_Rhodopsin-like_WP455_81793	4.70E-04	7	262	HRH1, BDKRB2, BDKRB1, F2RL2, PTGER2, CYSLTR2, GPR39
10	Hs_Focal_Adhesion_WP306_80308	5.11E-04	6	191	PDGFB, COL3A1, COL5A1, SPP1, THBS1, THBS2

Supple. Figure S2

a)

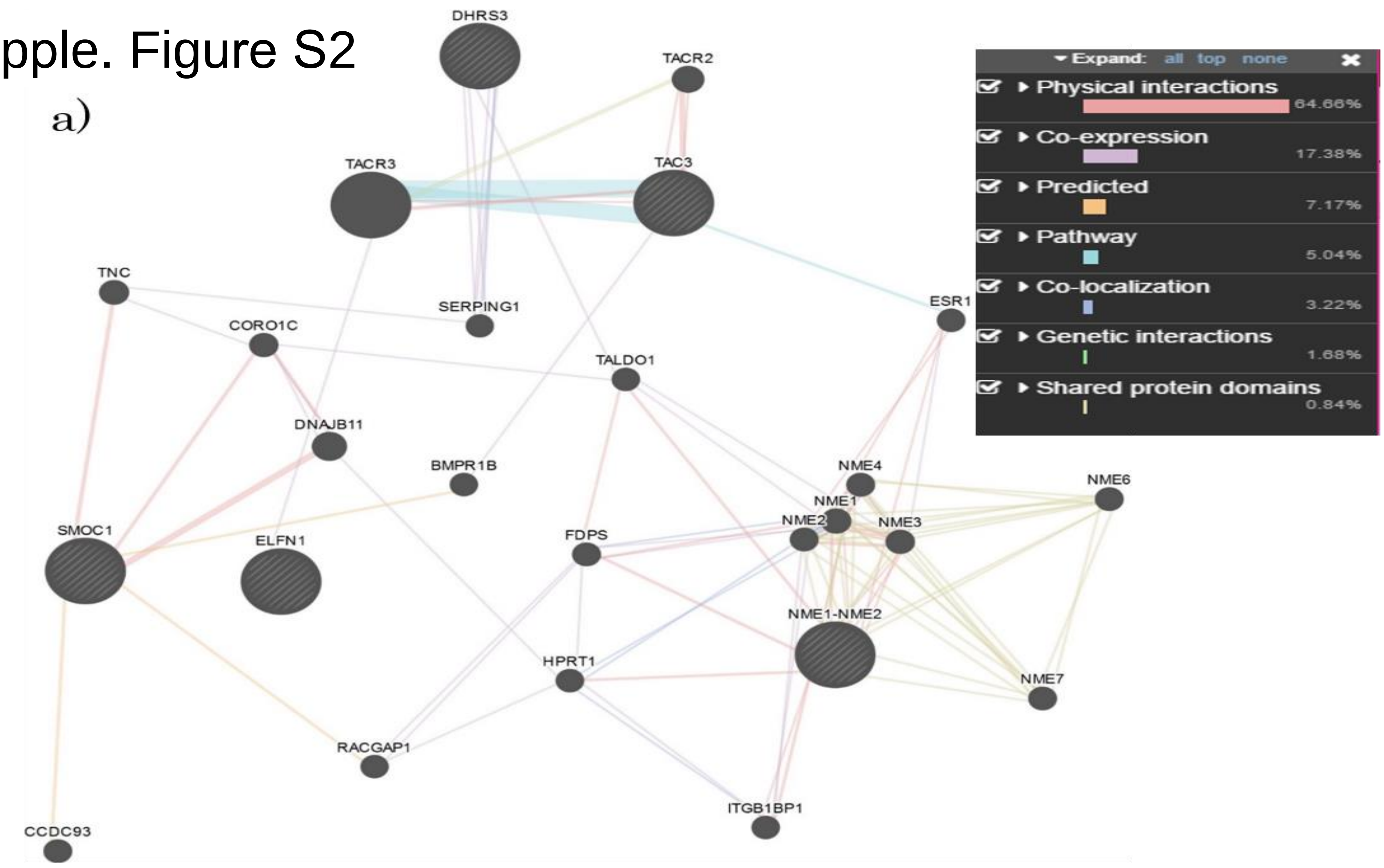

Gene and protein network of top 5 Up-regulated genes. Top 5 Up-regulated genes *(ELFN1, NME1-NME2, SOMC1, TAC3, DHRS3)* expressed in DHRS3-SK-N-SH cells, were analyzed by GENEMANIA online analysis tool. DHRS3 was not directly regulates other genes.

b)

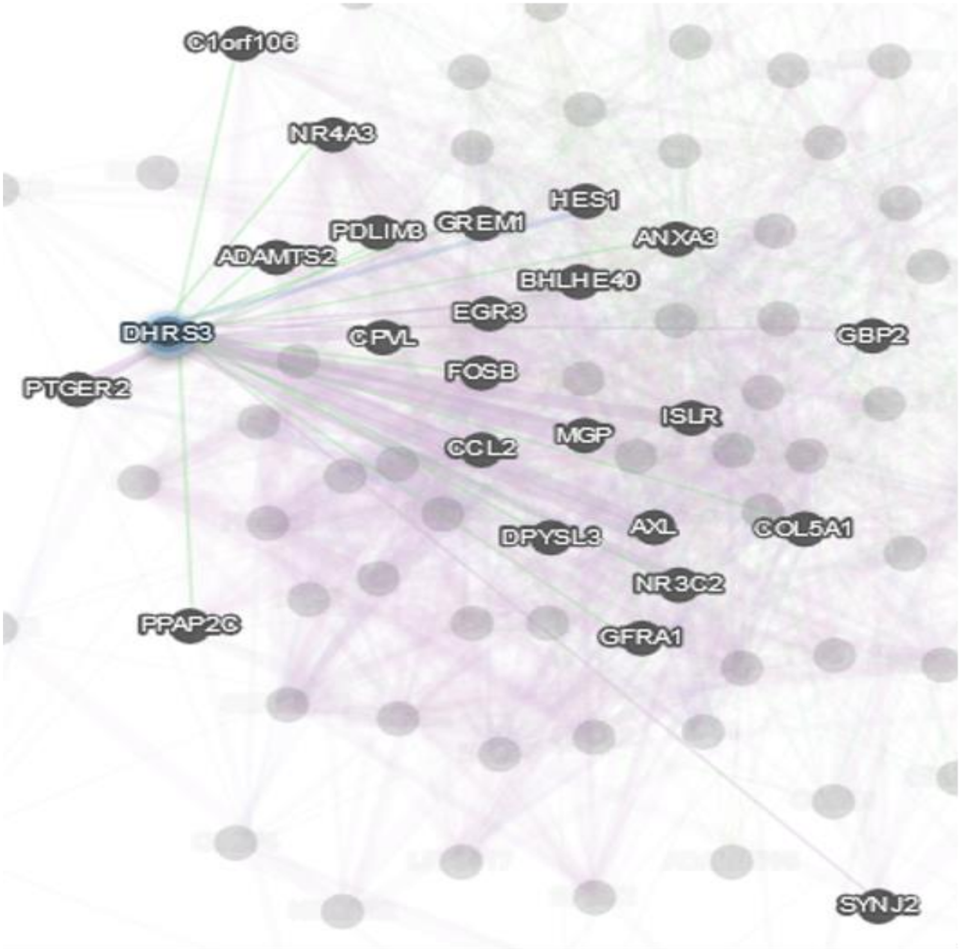

GENEMANIA network of directly interaction with DHRS3 of Up and Down-regulated genes (filtered by fold 10 alternation). Up-regulated 5 genes and down109 genes were analyzed. Analysis were performed by GENEMANIA.

c) **Top 29 of transcription factors Down-regulated in the SK-N-SH overexpress DHRS3 cells analyzed by Tfacts(P<0.05).**

Transcription Factor	P.value	E.value	Q.value	FDR control (B-H)	Interaction	Target genes
JUN	0.00E+00	0.00E+00	0.00E+00	9.09E-04	8	131
SMAD3	1.00E-05	5.50E-04	5.66E-05	1.82E-03	5	63
EGR1	4.00E-05	2.20E-03	1.51E-04	2.73E-03	5	91
SMAD2	1.00E-04	5.50E-03	2.83E-04	3.64E-03	3	21
GLI1	1.80E-04	9.90E-03	4.08E-04	4.55E-03	5	124
CTNNB1	2.80E-04	1.54E-02	5.29E-04	5.46E-03	7	306
SMAD4	9.60E-04	5.28E-02	1.55E-03	6.36E-03	3	45
TBP	1.31E-03	7.21E-02	1.86E-03	7.27E-03	3	50
STAT1	2.32E-03	1.28E-01	2.63E-03	8.18E-03	3	61
TCF7L2	2.32E-03	1.28E-01	2.63E-03	9.09E-03	3	61
NFKB1	3.15E-03	1.73E-01	3.24E-03	1.00E-02	4	141
WT1	6.13E-03	3.37E-01	5.79E-03	1.09E-02	2	27
ETS2	7.53E-03	4.14E-01	6.56E-03	1.18E-02	2	30
OLIG1	8.78E-03	4.83E-01	6.63E-03	1.27E-02	1	2
SMAD6	8.78E-03	4.83E-01	6.63E-03	1.36E-02	1	2
SP1	9.63E-03	5.30E-01	6.82E-03	1.46E-02	6	428
USF1	1.15E-02	6.31E-01	7.65E-03	1.55E-02	3	108
NOTCH1	1.31E-02	7.23E-01	8.22E-03	1.64E-02	2	40
PPARG	1.38E-02	7.58E-01	8.22E-03	1.73E-02	2	41
NFIC	1.65E-02	9.05E-01	8.61E-03	1.82E-02	2	45
NOTCH4	1.75E-02	9.61E-01	8.61E-03	1.91E-02	1	4
FOSL1	1.75E-02	9.61E-01	8.61E-03	2.00E-02	1	4
ID1	1.75E-02	9.61E-01	8.61E-03	2.09E-02	1	4
SP3	1.96E-02	1.08E+00	9.26E-03	2.18E-02	3	132
EPAS1	2.18E-02	1.20E+00	9.88E-03	2.27E-02	1	5
ESR1	2.32E-02	1.28E+00	1.01E-02	2.36E-02	2	54
SP4	3.04E-02	1.67E+00	1.28E-02	2.46E-02	1	7
CDX2	3.89E-02	2.14E+00	1.57E-02	2.55E-02	1	9
BCL6	4.31E-02	2.37E+00	1.69E-02	2.64E-02	1	10

DHRS3 CYP26A1 NROB1

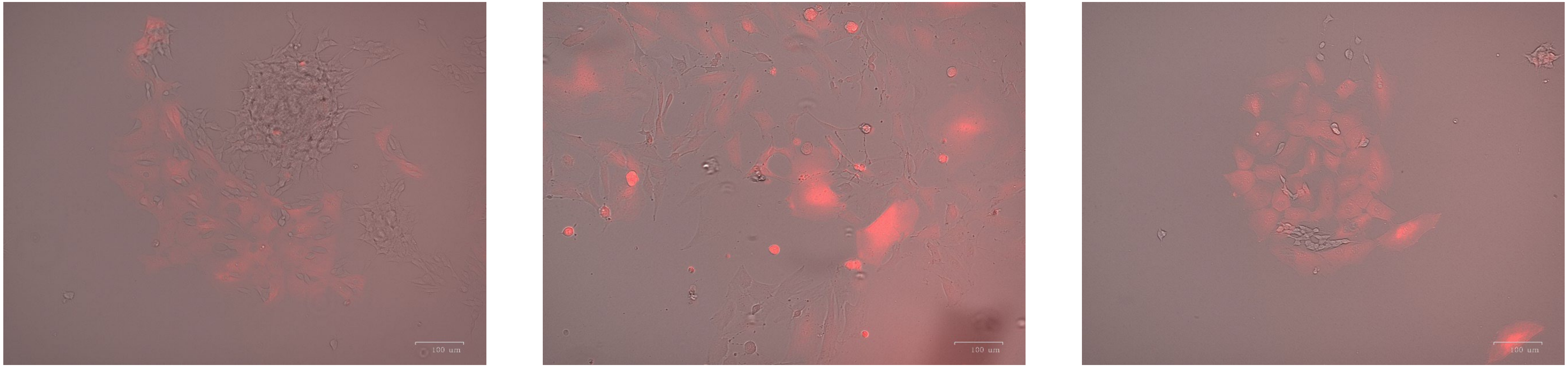

SK-N-SH Dox 5 days

Supple. Figure S 3 . Phenotypic alterations of SK-N-SH cells after Dox treatment for 5 days.

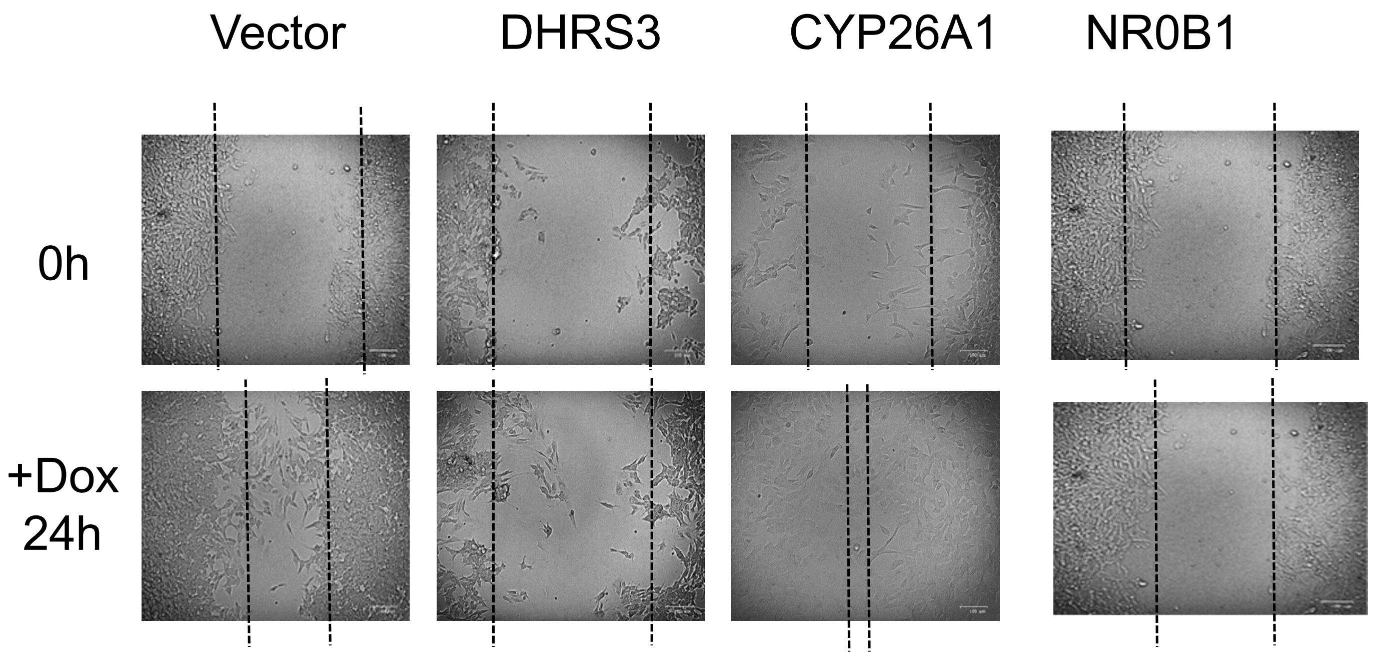

Supple. Figure S 4 . Wound healing migration assay of SK-N-SH cells.

Supple. Table S3. Cell sizes of transfected SK-N-SH cells in the flow-cytometry.

Gene	DHRS3		CYP26A1		NROB1	
Induction	Dox-	Dox+	Dox-	Dox+	Dox-	Dox+
FSC-A Median	87.38	190.18	103.12	173.13	71.16	197.43
SSC-A Median	44.33	128.71	106.04	111.44	42.33	125.11
